# Redox regulation of muscle adaptations to contractile activity and aging

**DOI:** 10.1152/japplphysiol.00760.2014

**Published:** 2015-03-19

**Authors:** Malcolm J. Jackson

**Affiliations:** MRC-Arthritis Research UK Centre for Integrated Research into Musculoskeletal Ageing (CIMA), Department of Musculoskeletal Biology, Institute of Ageing and Chronic Disease, University of Liverpool, Liverpool, United Kingdom

**Keywords:** reactive oxygen, nitric oxide, muscle, contractions

## Abstract

Superoxide and nitric oxide are generated by skeletal muscle, and these species are increased by contractile activity. Mitochondria have long been assumed to play the primary role in generation of superoxide in muscle, but recent studies indicate that, during contractile activity, membrane-localized NADPH oxidase(s) rapidly generate(s) superoxide that plays a role in redox signaling. This process is important in upregulation of rapid and specific cytoprotective responses that aid maintenance of cell viability following contractile activity, but the overall extent to which redox signaling contributes to regulation of muscle metabolism and homeostasis following contractile activity is currently unclear, as is identification of key redox-sensitive protein targets involved in these processes. Reactive oxygen and nitrogen species have also been implicated in the loss of muscle mass and function that occurs with aging, although recent work has questioned whether oxidative damage plays a key role in these processes. A failure of redox signaling occurs in muscle during aging and may contribute to the age-related loss of muscle fibers. Whether such changes in redox signaling reflect primary age-related changes or are secondary to the fundamental mechanisms is unclear. For instance, denervated muscle fibers within muscles from aged rodents or humans appear to generate large amounts of mitochondrial hydrogen peroxide that could influence adjacent innervated fibers. Thus, in this instance, a “secondary” source of reactive oxygen species may be potentially generated as a result of a primary age-related pathology (loss of neurons), but, nevertheless, may contribute to loss of muscle mass and function during aging.

the editor-in-chief of the *Journal of Applied Physiology* invited this review to accompany the presentation of the 2014 Edward F. Adolph lecture to the Environmental and Exercise section of the American Physiological Society, a lecture entitled “30 Years of chasing radicals in muscle: Redox regulation of muscle adaptations to contractile activity and aging.” My plan is to present a personal (and hence undoubtedly biased) view of how this exciting field has developed over 30 years, the key achievements that have been made, and to discuss some of the difficulties involved in studying this area. Of necessity, this is not a comprehensive description of all that has been discovered and is inevitably incomplete, since the field continues to evolve rapidly and relevant data appear on a regular basis that impact on our understanding of the area. Three key topics will be covered to which our research group have contributed a significant number of publications: *1*) generation of reactive oxygen and nitrogen species in contracting skeletal muscle; *2*) roles of reactive oxygen species in skeletal muscle; and *3*) reactive oxygen species in muscle aging.

## GENERATION OF REACTIVE OXYGEN AND NITROGEN SPECIES IN CONTRACTING SKELETAL MUSCLE

It is well established that skeletal muscle fibers generate superoxide and nitric oxide (NO), and these parent molecules can be converted to several secondary reactive oxygen species (ROS) and reactive nitrogen species (RNS). Superoxide and NO are generated from various sources within muscle fibers, and superoxide (53, 76), hydrogen peroxide (90), and NO (3, 46) are released into the interstitial space of muscle fibers (or generated on the extracellular side of the muscle plasma membrane). Contractile activity has been shown to increase the intracellular content or activities of superoxide, hydrogen peroxide, and NO (66, 75, 76, 84), while superoxide, hydrogen peroxide, hydroxyl radical, and NO have been detected in the muscle interstitial space (53, 67, 90).

**Fig. 1.**
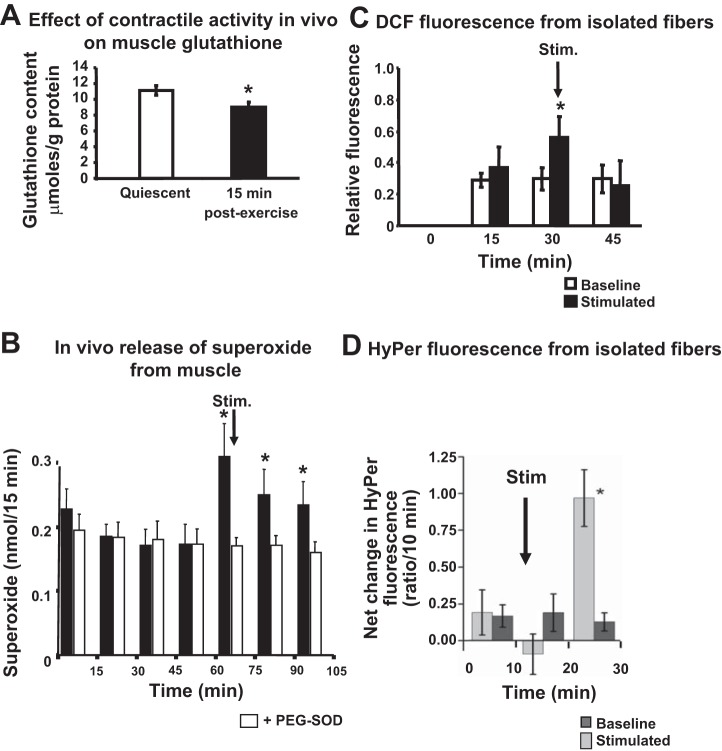
Examples of data derived from different approaches to study reactive oxygen species (ROS) generation in muscle or muscle fibers. *A*: reduction in glutathione content of muscles from wild-type mice in vivo following a 15-min period of isometric contractile activity. *Significant difference vs. quiescent. [Redrawn from Vasilaki et al. (90).] *B*: increase in interstitial superoxide monitored by microdialysis in the gastrocnemius muscle of mice during a 15-min period of isometric contractile activity. *Significant difference vs. polyethylene glycol (PEG)-superoxide dismutase (SOD). [Redrawn from Close et al. (11).] *C*: increase in intracellular 2′,7′-dichlorofluorescein (DCF) fluorescence from fibers isolated from the flexor digitorum brevis (FDB) muscle of mice and subjected to 15 min of isometric contractile activity in vitro. *Significant difference vs. baseline. [Redrawn from Palomero et al. (66).] *D*: increase in hydrogen peroxide content (indicated by increased *HyPer* fluorescence) in fibers isolated from the FDB muscle of mice and subjected to 10 min of isometric contractile activity in vitro. *Significant difference vs. baseline. [Redrawn from Pearson et al. (69).]

A number of different approaches have been used to demonstrate the increase in ROS that occurs during contractile activity. Although most data to date have been generated using nonspecific approaches, techniques have become increasingly sophisticated such that (for instance) new specific, genetically encoded fluorescent probes, such as *HyPer*, can report changes in single species in defined subcellular compartments (see Fig. 1 for examples of approaches that have been used). Much of the initial work in this area was based on the assumption that mitochondria were the main source of the ROS generated during contractile activity in muscle, but several recent publications disagree with this possibility (73). There is some debate about the precise location of NAD(P)H oxidase(s) that has been claimed as an alternative sources, but the presence of this enzyme in the skeletal muscle plasma membrane (41), sarcoplasmic reticulum (97), and the T-tubules (19) has been reported. The T-tubule localized enzyme appears to be particularly relevant, since it has been claimed to be specifically activated by contractions (19). In recent studies, we have examined the potential contribution of mitochondrial and nonmitochondrial sources to the acute increase in superoxide seen during muscle contractions (69, 79) and concluded that NADPH oxidase effects predominated over mitochondria during the short contraction periods (10–15 min) that were studied. Thus present data appear to indicate that a nonmitochondrial NADPH oxidase (likely to be the Nox2 isoform) is the major source of generation of superoxide during short-term contractile activity. The Nox4 isoform of NADPH oxidase has also been reported to be expressed in mitochondria and sarcoplasmic reticulum of skeletal muscle (79, 85), but any role in contraction-induced superoxide generation is unclear.

**Fig. 2.**
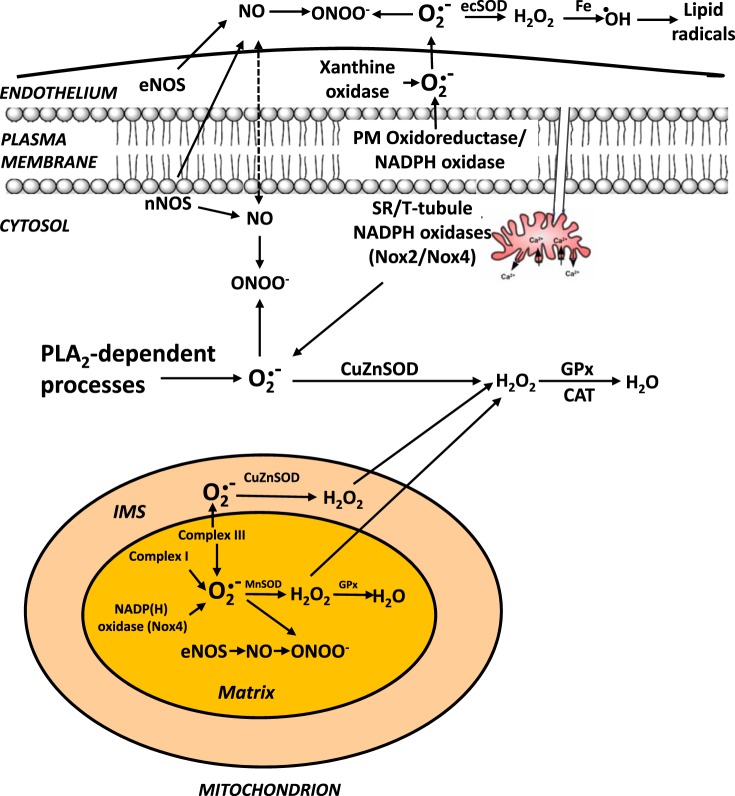
Updated working scheme for sites of ROS/reactive nitrogen species (RNS) generation by skeletal muscle demonstrating the potential role of Nox2 and Nox4 isoforms of NADPH oxidase in generating superoxide in mitochondria and cytosol and acknowledging the lack of evidence for any release of superoxide from mitochondrial during contractile activity. NO, nitric oxide; ONOO^−^, peroxynitrite; ecSOD, extracellular SOD; eNOS, endothelial NO synthase; nNOS, neuronal NO synthase; PLA_2_, phospholipase A_2_; GPx, glutathione peroxidase; CAT, catalase; IMS, intermembrane space. [Modified from Jackson (33).]

A number of specific ROS and RNS are detected in the extracellular space of skeletal muscle myotubes or isolated fibers in culture or in microdialysates from muscle interstitial fluid in vivo. It appears that muscle fibers may have generating systems for superoxide that release this species into the extracellular space (53, 76). Substantial diffusion of superoxide (or its protonated form) through the plasma membrane seems extremely unlikely (27), but other species that are detected in the muscle extracellular space (e.g., hydrogen peroxide and NO) can potentially diffuse across membranes and hence may originate from intracellular sites. Javesghani et al. (41) reported that a plasma membrane-localized NAD(P)H oxidase could release superoxide to the external face of the membrane, and Ward et al. (96) have described a stretch-activated NADPH oxidase (Nox2 isoform) that plays a major role in contraction-induced ROS generation in cardiac myocytes. This enzyme is also reported to be present in the skeletal muscle plasma membrane and appears to release superoxide to the outside of the cell. Other NAD(P)H-dependent systems have also been suggested to play a role in release of superoxide from muscle fibers (34). In muscle in vivo or intact muscle preparations ex vivo, xanthine oxidase enzymes in the endothelium may also play an important role in contraction-induced release of superoxide (24), and this enzyme has been claimed to be important in adaptations of muscle to contractile activity (22). Figure 2 summarizes our present understanding of the sites that have been identified for generation of ROS and NO in skeletal muscle fibers.

## ROLES OF ROS IN SKELETAL MUSCLE: OXIDATIVE DAMAGE OR REDOX SIGNALING?

**Fig. 3.**
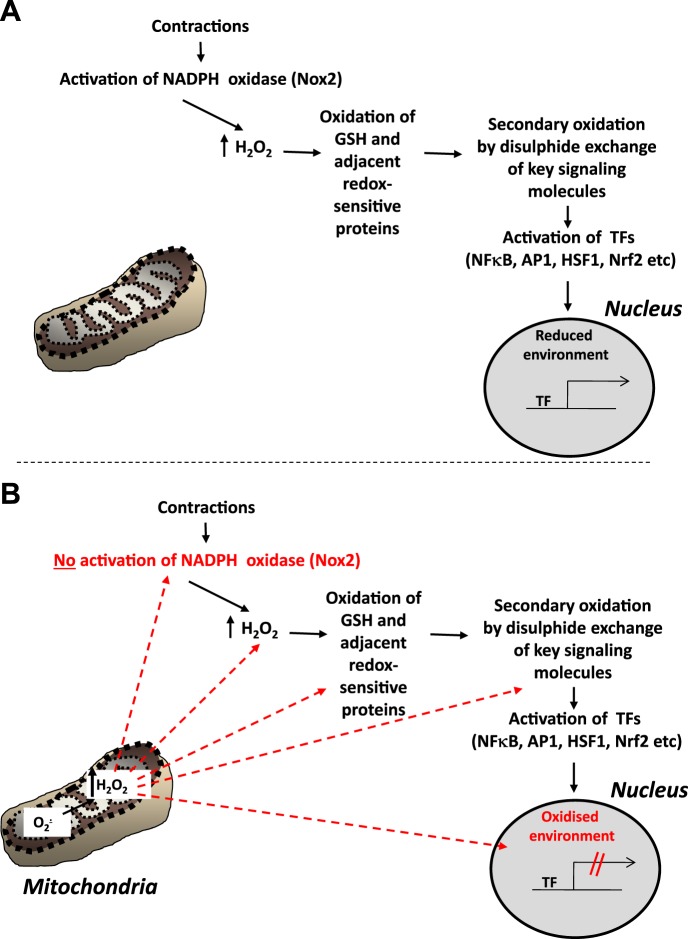
*A*: schematic representation of the redox signaling pathways that are postulated to lead to adaptive activation of transcription factors and upregulation of the expression of cytoprotective proteins following contractile activity in skeletal muscle. TF, transcription factor. [Redrawn and updated from Jackson and McArdle (35).] *B*: putative sites at which the redox signaling pathway may be modified in aging leading to a failure of adaptive responses to contractile activity. Excess hydrogen peroxide generated by mitochondria in the muscle during aging may influence the pathway shown in *A* at multiple points: prevention of activation of NADPH oxidase; a chronic increase in cytosolic hydrogen peroxide; aberrant chronic oxidation of glutathione and other redox sensitive signaling proteins; oxidation of the nuclear environment leading to a failure of TF to activate transcription. AP1, activator protein-1; HSF1, heat shock factor-1; Nrf2, nuclear transcription factor erythroid 2p45-related factor-2.

Although excess ROS can be deleterious to cells, causing oxidative damage to lipids, DNA and proteins (27), these species also appear to act as mediators of some adaptive processes following cellular stresses under normal physiological conditions. ROS mediate regulatory functions that lead to changes in cell and tissue homeostasis through modification of gene expression (17, 28, 36). Modification of specific thiol residues in proteins appears to be the major mechanism by which ROS exert such regulatory roles (40). Contractile activity increases the intracellular generation of superoxide and NO, and these species plus a number of secondary ROS and RNS (66, 73, 75) can mediate activation of a number of redox-regulated signaling pathways. The nature of these pathways has been the subject of extensive research, and redox-regulated processes (such as activation of NF-κB) have been shown to stimulate the expression of genes associated with myogenesis (2), catabolism, and mitochondrial biogenesis (4, 71, 87). Our group has been particularly interested in the role of ROS in activation of short-term cytoprotective changes in expression of regulatory enzymes and cytoprotective proteins in response to contractile activity (30, 53, 54). This appears to occur through redox-dependent activation of a number of transcriptional pathways, including the transcription factors, NF-κB, activator protein-1, heat shock factor-1 and nuclear transcription factor erythroid 2p45-related factor-2 (36, 42, 77, 91); see Fig. 3*A*.

## POTENTIAL MODULATING EFFECTS OF ANTIOXIDANT SUPPLEMENTS ON ROS-STIMULATED ADAPTATIONS TO CONTRACTILE ACTIVITY

Researchers have been attempting to suppress the presumed deleterious effects of ROS and RNS generated during exercise since the first descriptions of their generation in this situation (e.g., Ref. 15). There has been little evidence of beneficial effects on muscle from such interventions, but the realization that these species play important roles in redox signaling has prompted a rethink of what antioxidants might achieve in this situation. Our group initially demonstrated that high doses of vitamin C could inhibit rapid stress responses to acute exercise (45), and this line was pursued by others who reported that high doses of antioxidants could reduce the training effects of exercise on muscle mitochondrial biogenesis, maximal O_2_ uptake, and improvements in insulin sensitivity (23, 77). The implication of such studies is that ROS or RNS play a key role in regulating multiple training-induced adaptations to muscle in humans and animals. Unfortunately, such findings could not be repeated by other scientists, who reported normal adaptations to exercise training, despite administration of high-dose antioxidants (e.g., Ref. 29). This difference resulted in an intense head-to-head debate in the scientific literature from the groups reporting these differing results (e.g., Ref. 31). There are a number of differences in experimental design that are likely to underlie the differences in reported outcomes, including the study of animals or humans, trained or untrained subjects, the durations and protocols for the training, the choice of markers of oxidative stress, the time points studied, the use of muscle vs. blood markers, and many more potential factors. A recent article by Paulsen et al. (68) has shed some light on this controversy, although this also illustrates the complexity of relating signaling processes to true physiological function. The study appears to confirm that these supplements do not universally inhibit major physiological adaptations to exercise training, although they did inhibit potentially relevant changes in mitochondrial proteins. A full explanation for the apparent discrepancies in the literature in this area is unlikely to appear until more is known about the scope and importance of redox signaling in muscle, but the present debate highlights the potential unintended consequences of untargeted use of high-dose antioxidant supplements.

## ROS AND MUSCLE AGING

Aging leads to a reduction in muscle mass and function that contributes to physical instability and increased risk of falls (99), such that, by the age of 70 yr, skeletal muscle cross-sectional area has declined by 25–30% and muscle strength by 30–40% (72). In both humans and rodents, there is evidence that the age-related reduction in muscle mass and function is primarily due to decreased numbers of muscle fibers, and atrophy and weakening of the remaining fibers (6, 49, 50), although a recent study suggests atrophy of type II fibers without fiber loss is the major contributor to the decreased muscle mass seen in healthy elderly human subjects (61). Most of the intrinsic and extrinsic changes regulating muscle aging in humans have been observed in rodents, indicating that mice and rats can provide relevant models of human sarcopenia (14). Denervation also contributes to loss of muscle mass in humans and rodents (13, 39). The comparable changes in morphology seen in myofibers of aged rodents and humans suggest the mechanisms leading to muscle loss and atrophy at the cellular level are comparable (57). Muscle from old rodents also shows an increased proportion of more oxidative fibers (13) and an attenuation of various responses to contractile activity, including acute stress responses (91), mitochondrial biogenesis (51), and the contraction-induced increase in muscle protein synthesis (12). These are potentially important aspects of the multiple age-related deficits in muscle, including contributing to slowed reactions and an inability to fine-tune movements, while transgenic studies indicate that correction of specific attenuated responses to contractions can preserve muscle force generation in aged mice (7, 44, 52).

## OXIDATIVE DAMAGE AND DEFECTIVE REDOX SIGNALING IN MUSCLE FROM OLD MICE AND HUMANS

An increase in oxidative damage has been reported in tissues (including skeletal muscle) of all aged organisms compared with levels found in young organisms (16, 81, 90). The possibility that increased oxidative damage plays a key role in age-related tissue dysfunction has received considerable attention. In nonmammalian models, interventions designed to reduce the activities of ROS, such as overexpression of CuZn, superoxide dismutase 1 (SOD1), catalase or both in *Drosophila* (63–65), or treatment with a MnSOD and catalase mimetic in *C. elegans* (56), extended lifespan and thus support the hypothesis, but these effects have not been confirmed in other studies (20). In mammals, only a small number of manipulations designed to reduce ROS activities and/or oxidative damage have increased lifespan (82, 98). It, therefore, appears that increased ROS generation is not the fundamental cause of aging (or more precisely, the fundamental determinant of lifespan). Many studies have reported that mitochondrial ROS generation is increased in skeletal muscle during aging (Refs. 55, 88 for reviews) in association with impaired function and oxidative damage to mitochondrial components (38, 81). Furthermore, other studies indicate that interventions to reduce mitochondrial hydrogen peroxide content (82) or increase cytoprotective proteins that reduce oxidative damage (7) can preserve muscle function during aging. Increased mitochondrial ROS generation has also been proposed to play a key mediating role in pathological changes in muscle in conditions such as disuse atrophy (74).

## MODIFICATION OF MUSCLE ROS DURING AGING: KNOCKOUT OF KEY REGULATORY PROTEINS

A number of studies have examined the effects of deletion of regulatory enzymes for ROS, but, despite frequent observation of increased oxidative damage in these models, no clear relationship with skeletal muscle aging was seen (38). The exception to this pattern was in mice with a whole body deletion of SOD1, which show neuromuscular changes with aging that appear to reflect an accelerated skeletal muscle aging process (58). Adult SOD1 knockout (*SOD1KO*) mice show a decline in skeletal muscle mass, loss of muscle fibers and a decline in the number of motor units, loss of motor function and contractility, partial denervation, and mitochondrial dysfunction by 8 mo old (37, 47, 92). The fiber loss in *SOD1KO* mice is accompanied by degeneration of neuromuscular junctions (NMJs) (37). These changes are also seen in old wild-type (WT) mice, but not until after 22 mo of age. Hence we have proposed that *SOD1KO* mice are a useful model to examine the potential role of ROS in skeletal muscle aging (32).

It is relevant to consider why only the *SOD1KO* mice show an accelerated muscle aging phenotype, although other models with knockout of regulatory enzymes for ROS or RNS also show an increase in oxidative damage to muscle. SOD1 is expressed in both the cytosol of cells and within the mitochondrial intermembrane space, where it is likely to be present at high concentration compared with cytosolic SOD1 (43). One implication of this is that lack of SOD1 may influence redox homeostasis in the mitochondria, in addition to the cytosol, and hence that disturbances in either cytosolic or mitochondrial redox may underlie the accelerated skeletal muscle aging phenotype seen in *SOD1KO* mice. In our studies, we examined the nature of the reactive species that are generated in mice lacking SOD1. Some studies of aging models have suggested that the decline in tissue function that occurs with aging and the accelerated loss of skeletal muscle fibers in *SOD1KO* mice may be caused by superoxide toxicity (38, 56). An alternative possibility is that superoxide and NO may react chemically to form peroxynitrite, a reaction that competes with the dismutation of superoxide to hydrogen peroxide by SOD (5). In adult SOD1 null mice, the phenotype may, therefore, be associated with excess superoxide, but may also be due to increased peroxynitrite or a reduction in NO bioavailability. We demonstrated that, similar to muscle fibers from old WT mice, those from adult SOD1 knockout mice showed an increase in oxidation of the nonspecific intracellular ROS probe, 2′,7′-dichlorodihydrofluorescin-diacetate (DCFH) at rest compared with fibers from adult WT mice (92). Surprisingly, the fibers from *SOD1KO* mice showed no increase in DCFH oxidation following contractile activity, although an increase in DCFH oxidation was seen in muscle fibers from adult WT mice following contractile activity. The explanation for this is currently unclear, although DCFH is relatively insensitive to oxidation by superoxide, but is oxidized by other ROS, including hydrogen peroxide, hydroxyl radicals, peroxynitrite, and NO (60). Single muscle fibers from flexor digitorum brevis of WT and *SOD1KO* mice were, therefore, also loaded with NO-sensitive [4-amino-5-methylamino-2′,7′-difluorofluorescein diacetate (DAF-FM)] and superoxide-sensitive [dihydroethidium (DHE)] probes (78). These studies illustrated that a lack of SOD1 in the fibers from *SOD1KO* mice did not increase superoxide availability at rest, since no increase in ethidium or 2-hydroxyethidium formation from DHE was seen in fibers from *SOD1KO* mice compared with those from WT mice. Fibers from *SOD1KO* mice were found to have decreased NO availability (decreased DAF-FM fluorescence), increased 3-nitrotyrosines in muscle proteins, indicating increased peroxynitrite formation, and increased content of peroxiredoxin V (a peroxynitrite reductase) compared with WT mice. Following contractile activity, muscle fibers from *SOD1KO* mice also showed substantially reduced generation of superoxide compared with fibers from WT mice. Inhibition of NO synthase to reduce NO availability, and hence the potential for formation of peroxynitrite, did not affect DHE oxidation in fibers from WT or *SOD1KO* at rest or during contractions. In contrast, fibers isolated from neuronal NO synthase transgenic mice showed increased DAF-FM fluorescence and reduced DHE oxidation in resting muscle fibers. These data appear to indicate that peroxynitrite is formed in muscle fibers as a consequence of lack of SOD1 in *SOD1KO* mice and may, therefore, contribute to fiber loss in this model. More generally, these data also support the hypothesis that NO regulates superoxide availability and peroxynitrite formation in muscle fibers (78).

## RELATIVE ROLE OF A LACK OF SOD1 IN MUSCLE OR IN MOTONEURONS IN THE ACCELERATED AGING PHENOTYPE SEEN IN SOD1KO MICE.

To specifically examine how changes in muscle SOD1 might influence age-related changes in muscle, mice with muscle-specific deletion of SOD1 (*mSOD1KO* mice) were examined (100), but these mice show no evidence of premature NMJ degeneration or loss of muscle fibers and surprisingly showed some muscle hypertrophy (100). We examined whether the changes in ROS generation observed in the global knockout model (*SOD1KO* mice) were also seen in *mSOD1KO* mice. In brief, the multiple changes in markers of oxidative damage and adaptation seen in *SOD1KO* mice and described above were not observed in the *mSOD1KO* mice, including no evidence for the increases in 3-nitrotyrosines and peroxiredoxin V previously reported in muscles of *SOD1KO mice* (79, 100).

To determine the role of motoneurons in the loss of muscle mass and function seen in *SOD1KO* mice, a transgenic *SOD1KO* mouse in which human SOD1 is expressed in neurons under control of a synapsin 1 promoter (*nSOD1Tg-SOD1KO* mice) was established (80). These “nerve rescue” mice expressed SOD1 in central and peripheral neurons, but not other tissues. Sciatic nerve CuZnSOD content in *nSOD1Tg-SOD1KO* mice was ∼20% that of WT control mice, but they showed no loss of muscle mass or maximum isometric specific force production at 8–12 mo of age, when significant reductions were seen in *SOD1KO* mice (80). Thus these data appeared to demonstrate that at least 20% of WT CuZnSOD levels in neurons is essential in preserving skeletal muscle and NMJ structure and function in *SOD1KO* mice and implicated a lack of SOD1 specifically in motoneurons in the pathogenesis of the accelerated muscle aging phenotype seen in the whole body SOD1 null mice.

Adult mice lacking SOD1, therefore, replicate many of the features seen in old WT mice, and it appears that further examination of this model and variants of the model with tissue-specific modification of SOD1 content could identify key mechanisms leading to loss of muscle fibers and function that are relevant to aging of WT mice. The initiating role for the motoneuron in this model provides a means of determining mechanisms by which disruption of redox homeostasis in the motoneuron can cause loss of muscle fibers, and we speculate that this may also be important for aging in WT mice. Although *SOD1KO* mice are a model in which fundamental questions about mechanisms that are highly relevant to understanding muscle aging can be addressed, it is reiterated that there is no evidence that a simple lack of SOD1 contributes to aging-related loss of muscle in WT mice or humans.

## POTENTIAL PRIMARY AND SECONDARY SOURCES OF ROS DURING AGING

Increased ROS generation by mitochondria has been implicated in aging of muscle and other tissues for a considerable period of time. This process was originally claimed to have a primary role in the aging process (38, 81), but the recent work of Pérez et al. (70) and Gems and Doonan (20) argues strongly against a primary role for oxidative damage in skeletal muscle in aging. Other recent data also indicate that not all mitochondria isolated from aging muscle show increased ROS generation (21, 25). Despite these contrasting data, some interventions that specifically reduce mitochondrial ROS (mice overexpressing catalase in mitochondria, mCAT mice; Ref. 82) or protect against oxidative damage (mice overexpressing heat shock protein 10, HSP10^Tg^ mice; Ref. 44) appear to preserve muscle mass and function. We have previously proposed that excess generation of hydrogen peroxide by mitochondrial from aged mice could act to attenuate the ability of muscle fibers from aged mice to adapt to contractile activity (35) as shown schematically in Fig. 3*B*. It is, therefore, relevant to consider whether increased mitochondrial ROS might play a secondary role in aging processes and be a consequence of more direct effects of aging. Potential examples of this may be the increase in muscle mitochondrial ROS that appears to occur secondarily to other age-related changes in the *SOD1KO* mouse studies described above, and also by the observation that experimental denervation leads to a very large sustained increase in muscle mitochondrial ROS generation (59). Data from both of these situations support the possibility that functional denervation of individual muscle fibers may lead to a fiber-specific increase in mitochondrial ROS generation.

There is extensive evidence that some denervation of muscle fibers occurs with aging. In humans, an ∼25% reduction in the number of motoneurons occurs with aging, and, although the causes of this loss are unknown, small motoneurons (which tend to innervate type I fibers) are preserved relative to large motoneurons. Over time, the loss of large motoneurons appears to be partially compensated by a sprouting phenomenon through which small motoneurons innervate those type II fibers that have become temporarily denervated, and hence these fibers acquire a slower phenotype. This process is thought to be incomplete and eventually the new “giant” motor units are lost (13). Studies to determine whether the age-related loss of muscle fibers is associated with loss of motor units in humans and rodents indicate that substantial net loss of whole motor units occurs with increasing age in both species (8, 18, 48). Atrophy and loss of axons has been reported in older individuals (93), together with additional abnormalities in peripheral nerves, including segmental demyelination (1, 83), swollen demyelinated and remyelinated axons, and denervated Schwann cell columns (26). A variety of changes have been reported in NMJs of aged mice, including axonal swelling and sprouting, withdrawal of axons from postsynaptic sites, and fragmentation of the postsynaptic structures (10, 86), and there is evidence from older postmortem studies that such changes are seen in elderly humans (62). Recent data from rodents also indicate that, despite the loss of peripheral axons that occurs with aging, the number of motoneuron cell bodies in the lumbar spinal cord are unchanged, suggesting that changes may predominantly occur in peripheral regions of motor units (10). Thus it appears that motor axon and NMJ loss with aging occurs in parallel with loss of muscle fibers and diminished muscle function (9, 49, 50) in both humans and animals, but it is currently unclear whether either of these is the primary event (48, 95).

Thus we speculate that a feasible integrating mechanism based on the present data relating to the age-related changes in ROS activities and redox signaling in muscle is that denervation of individual muscle fibers leads to a large increase in mitochondrial ROS generation in the affected fibers. Since the key ROS generated in mitochondria of the denervated fibers appears to be hydrogen peroxide or other peroxides, such species are membrane permeable and could diffuse to adjacent innervated fibers, leading to redox-related changes in oxidative damage and redox signaling.

## CONCLUSIONS

In conclusion, recent data indicate that membrane-localized NADPH oxidase(s) are the source of the superoxide generated in skeletal muscle during contractile activity that play an important role in redox signaling, and that these pathways upregulate cytoprotective responses that aid maintenance of cell viability following contractile activity. A failure of this redox signaling pathway appears to occur in muscle during aging and may contribute to the loss muscle fibers, but whether these changes are primary or secondary events in aging is unclear. One possible explanation that provides an explanation for the present data is that a small number of denervated muscle fibers within the muscle may generate large amounts of hydrogen peroxide from mitochondria, and that this can influence redox signaling in adjacent innervated fibers, and thus provides a secondary source of ROS that may contribute to loss of muscle mass and function during aging.

## GRANTS

This work has also been supported by many funding agencies, including the Biotechnology and Biological Sciences Research Council, Medical Research Council, Arthritis Research UK, Research into Ageing, Wellcome Trust, and US National Institute on Aging.

## DISCLOSURES

No conflicts of interest, financial or otherwise, are declared by the author(s).

## AUTHOR CONTRIBUTIONS

Author contributions: M.J.J. conception and design of research; M.J.J. performed experiments; M.J.J. analyzed data; M.J.J. interpreted results of experiments; M.J.J. prepared figures; M.J.J. drafted manuscript; M.J.J. edited and revised manuscript; M.J.J. approved final version of manuscript.
